# High Resolution and Labeling Free Studying the 3D Microstructure of the Pars Tensa-Annulus Unit of Mice

**DOI:** 10.3389/fcell.2021.720383

**Published:** 2021-10-08

**Authors:** Jian-Ping Wu, Xiaojie Yang, Yilin Wang, Ben Swift, Robert Adamson, Yongchang Zheng, Rongli Zhang, Wen Zhong, Fangyi Chen

**Affiliations:** ^1^Academy of Advanced Interdisciplinary Studies, Southern University of Science and Technology, Shenzhen, China; ^2^Department of Biomedical Engineering, Southern University of Science and Technology, Shenzhen, China; ^3^Core Research Facilities, Southern University of Science and Technology, Shenzhen, China; ^4^College of Computing, Australian National University, Canberra, ACT, Australia; ^5^School of Biomedical Engineering, Electrical and Computer Engineering, Dalhousie University, Halifax, NS, Canada; ^6^Peking Union Medical College Hospital, Chinese Academy of Medical Sciences and Peking Union Medical College, Beijing, China; ^7^Guangdong Provincial People’s Hospital, Guangdong Academy of Medical Science, School of Medicine, South China University of Technology, Guangzhou, China; ^8^School of Mechanical Engineering and Automation, Xihua University, Chengdu, China; ^9^Department of Biology, Brain Research Centre, Southern University of Science and Technology, Shenzhen, China

**Keywords:** tympanic membrane, pars tensa-annulus unit, 3D microstructure, collagen, elastic fibres

## Abstract

Hearing loss is a serious illness affecting people’s normal life enormously. The acoustic properties of a tympanic membrane play an important role in hearing, and highly depend on its geometry, composition, microstructure and connection to the surrounding annulus. While the conical geometry of the tympanic membrane is critical to the sound propagation in the auditory system, it presents significant challenges to the study of the 3D microstructure of the tympanic membrane using traditional 2D imaging techniques. To date, most of our knowledge about the 3D microstructure and composition of tympanic membranes is built from 2D microscopic studies, which precludes an accurate understanding of the 3D microstructure, acoustic behaviors and biology of the tissue. Although the tympanic membrane has been reported to contain elastic fibers, the morphological characteristic of the elastic fibers and the spatial arrangement of the elastic fibers with the predominant collagen fibers have not been shown in images. We have developed a 3D imaging technique for the three-dimensional examination of the microstructure of the full thickness of the tympanic membranes in mice without requiring tissue dehydration and stain. We have also used this imaging technique to study the 3D arrangement of the collagen and elastic fibrillar network with the capillaries and cells in the pars tensa-annulus unit at a status close to the native. The most striking findings in the study are the discovery of the 3D form of the elastic and collagen network, and the close spatial relationships between the elastic fibers and the elongated fibroblasts in the tympanic membranes. The 3D imaging technique has enabled to show the 3D waveform contour of the collagen and elastic scaffold in the conical tympanic membrane. Given the close relationship among the acoustic properties, composition, 3D microstructure and geometry of tympanic membranes, the findings may advance the understanding of the structure—acoustic functionality of the tympanic membrane. The knowledge will also be very helpful in the development of advanced cellular therapeutic technologies and 3D printing techniques to restore damaged tympanic membranes to a status close to the native.

## Introduction

The tympanic membrane (TM) receives and converts sound-pressure waves over a broad frequency spectrum from the external environment into mechanical vibrations, which are conducted through the ossicular chain resulting in movement of perilymph and neural transduction in the hair cells of cochlea (Decraemer et al., 1991; Caminos et al., 2018). Damage, perforation and degeneration of the TM due to injury, otitis media and aging are leading causes of hearing impairment and deafness, with obvious impacts on an individual’s life quality.

**FIGURE 1:**
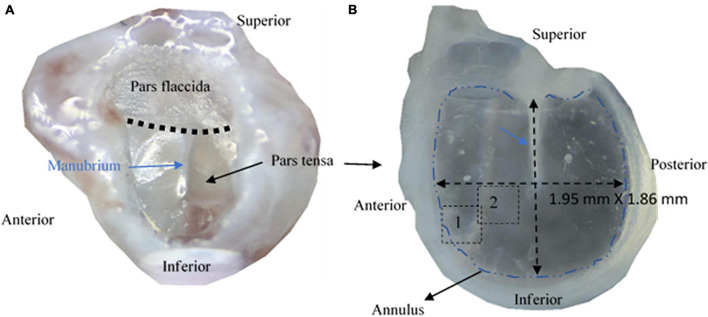
Photographs of tympanic membranes of mice. **(A)** A tympanic membrane of a mouse is typically made of pars flaccida and pars tensa. **(B)** The pars tensa attached with the anulus was extracted for studying the 3D microstructure using multiphoton microscopy.

In mammals, the TM appears as a shallow conical dish with the apex toward the medial side. It consists of two distinctive parts known as the pars flaccida and pars tensa (Lim, 1968, 1970, 1995), as shown in Figure 1A. Across the thickness, a TM comprises three distinctive layers; the external epidermal layer, the fibrous lamina propria and internal mucosal layer (Igarashi and Kawamata, 1993; Lim, 1995). The fibrous lamina propria, which is composed predominantly of collagen fibers, largely determines the shape and mechanical behaviors of the TM (Secondi, 1951; Funnell and Laszlo, 1982; Lim, 1995; Stenfeldt et al., 2006). The lamina propria of the pars flaccida is made of loose collagen (Lim, 1995). In contrast, the lamina propria within the pars tensa consists of collagen fibers organized into radial and circumferential arrays, respectively, in the lateral and medial side of the TM (Uno, 2000; Fay et al., 2006; Jackson et al., 2008; Liu et al., 2016). Despite extensive research, the unique arrangement of the collagen fibers in the physiology of TMs remains exclusive. TMs also contain a small fraction of elastic fibers (Chole and Kodama, 1989; Lim, 1995; Volandri et al., 2011) but the microstructural features of the elastic fibers and the spatial relationship of the elastic fibers with the cells, collagen fibers and capillaries have not yet been shown in images.

While physically coupling to the manubrium of the malleus in the ossicular chain and anchoring itself on the cartilaginous annulus of the bony tympanic ring attached to the bulla, the pars tensa makes up the majority of the TM (De Greef et al., 2016). The morphology, composition and microstructure of the pars tensa as well as its connection to the annulus are considered to play a crucial role in the acoustic function of TMs (De Greef et al., 2014; Kozin et al., 2016). Although the conical shape of TMs is critical to the sound propagation of the TMs (Decraemer et al., 1991; Fay et al., 2006), it creates significant impediment to study the 3D microstructure and composition of the TMs using conventional 2D microscopy.

Various technologies have been developed to study the morphology, composition and microstructure of TMs. Conventional histology is routinely used in identifying the pathological changes of TMs at a cellular level (Stenfeldt et al., 2006). However, the use of traditional optical microscopy with insufficient imaging resolution restricts the application of the technique from revealing the ultrastructure of the collagen fibers and subtle microstructural change of TMs beyond a subcellular level. Alternatively, the electron microscopy (EM) has superior imaging resolution for tracing the ultrastructure of the TMs (Igarashi and Kawamata, 1993; Lim, 1995; Uno, 2000) but it requires an extreme imaging environment, which could denaturize the form of the TMs and potentially introduce artifacts. Besides, none of these technologies is capable of imaging the microstructure and composition of the brittle and conical TMs. To date, knowledge about the composition and 3D microstructure of TMs has primarily been built from studies using 2D imaging technologies. This has prevented deep understanding of the 3D microstructure, acoustic properties and biology of TMs.

Multiphoton microscopy (MPM) offers submicron imaging resolution for studying three-dimensionally the intrinsic microstructure of biological tissues without tissue stain, dehydration and sectioning (He et al., 2013c; Pang et al., 2017; Wu et al., 2017). MPM harnesses non-linear optical imaging technologies that detect the photons emitted from featured molecular compounds under the laser excitation at the near-infrared spectrum. This guarantees that the emitted photons only occur at the focal plane for 3D imaging the microstructure of biological tissues without a pinhole. Second harmonic generation (SHG) and two-photon fluorescence (TPF) imaging are the two primary imaging modalities used in modern MPM. The SHG signals arise from non-absorptive tissue-light interaction of substances possessing a non-centrosymmetrical and crystallized molecular structure (Prockop and Kivirikko, 1995), such as collagen, which produces a large quantity of SHG signals under two-photon excitation for SHG imaging without photobleaching (Campagnola and Loew, 2003; Cox et al., 2003; Chen et al., 2012; Wu et al., 2017).

In comparison, TPF utilizes two-photon excitation to generate the endogenous fluorescence of tissues (Cahalan et al., 2002; Koch et al., 2014). One of the important applications of TPF in biology is to study the elastic fibers of tissues through the autofluorescence emission of the fluorophores presenting in the elastin under two-photon excitation (König et al., 2005; Mansfield et al., 2009; He et al., 2013b, c, 2014). By comprising a central core of elastin attached by microfibrils, elastic fibers endow the tissues with the elasticity required to recover quickly from deformation to eliminate fatigue damages, while also influence the biological function and physiology of the tissues (Montes, 1996; Debelle and Tamburro, 1999). Elastin is well present in cell membranes, the walls of blood vessels and extracellular matrix (ECM) of many tissues such as lungs, skin, tendons and cartilage (Curran et al., 1993; Li et al., 1998; He et al., 2013a, b; Pang et al., 2017). Therefore, TPF is an ideal tool for visualizing cells and capillaries without tissue dehydration and stain (Zheng et al., 2011). Numerous studies have applied SHG and TPF for examining the spatial network of collagen with the elastic fibers and cells without tissue dehydration and stain (Zoumi et al., 2004; Mansfield et al., 2009; He et al., 2013a,b,c; Pang et al., 2017).

Using SHG and TPF, we have developed a 3D imaging technique for label-free imaging of the 3D microstructure of hydrated and full-thickness TMs in mice. The study has led to reveal the intrinsic collagen structure and the spatial arrangement of the collagen with the elastic fibers, fibroblasts, vimentin positive cells, capillaries and chondrocytes in the pars tensa-annulus unit. The high-resolution images of the collagen fibers have also provided the opportunity to perform quantitative analysis of the orientation of the collagen fibers comprising the pars tensa for a consistent description of the collagen orientation.

We believe this is the first study that has characterized simultaneously the elastic fibrillar network and the intrinsic microstructure of the collagen with the elastic fibers, cells and capillaries in the pars tensa-annulus unit at a status close to the native. This study has advanced our knowledge about the 3D microstructure and composition of the pars tensa-annulus unit and the connection between the pars tensa and annulus. The knowledge will be useful to the understanding of the acoustic function and biology of TMs, and development of advanced cellular therapy and 3D printing technologies to restore perforated TMs to an extension close to the native.

## Materials and Methods

### Samples

*Ex vivo* mouse TM samples used in this study were donated by other unrelated research in accordance with approvals from the Animal Ethics Committee at Southern University of Science and Technology, Shenzhen, China. A total of five bullae with a normal appearance were harvested from five C57 mice (two females, three males) of 10–12 weeks old. The bullae were washed thoroughly with neutral phosphate buffered solution (PBS) and fixed in 4% paraformaldehyde for 24 hours before being decalcified in 10% ethylene diamine tetra acetic acid (EDTA) (Phygene Biotechnology, Fuzhou, China) for 48 hours. After washing the bullae with neutral PBS, with the aid of a stereoscope, the TMs were dissected from the bullae with the cartilaginous annulus attached (Figure 1) before the epidermal and mucosal layers of the TMs were carefully removed from the TMs using a surgical knife and tweezers. Only the lamina propria without visible fiber broken under the stereoscope was used as the sample.

After cleaning with PBS, the samples were carefully placed on a glass slide with the medial side up and coated a drop of PBS before covered with a coverslip for SHG and TPF imaging. During imaging, the tissues were kept in a hydrated status.

### Imaging Acquisition and Thickness Measurement of Collagen Fibers and Fibrils

An Olympus FVMPE-RS upright MPM (Olympus, Japan) with an apochromatic 25 × /NA 1.05 water-immersion objective lens offering a 2.0 mm working distance (Olympus, Japan) was used in this study. The MPM is equipped with a femtosecond-pulsed Ti:Sa laser (Mai Tai DeepSee, Spectral-Physics, United States) with a tunable wavelength range from 690 to 1,300 nm.

The SHG and TPF imaging were performed by tuning the excitation laser at 890 nm and fitting a filter cube (Chroma Technology, China) with a 475 nm dichroic mirror, a green barrier filter of 495–540 nm and a violet barrier filter of 410–455 nm. In this configuration, the emission light was split into two independent imaging channels for acquiring green-fluorescence and SHG image stacks through the photomultiplier tubes (PMT). The resultant green fluorescence channel at the emission wavelength of 495–540 nm was assigned for TPF imaging. The violet imaging channel was set up in a reflectance mode and used for acquiring SHG images at 445 nm, which is exactly half the wavelength of the excitation.

For studying the collagen and elastic fibers within the pars tensa-annulus unit, the SHG and TPF imaging stacks were acquired simultaneously at pixel resolution of 1,024 × 1,024 over the field of the view of about 509 μm × 509 μm. High magnification SHG imaging stacks at the field of the view of 127 μm × 127 μm were also acquired at the pars tensa (the dashed square shown in Figure 4A) to verify that the coast collagen fibers or bundles constituting the pars tensa were composed of the subclass collagen fibrils (e.g., Figure 7). The imaging step used to acquire the imaging stacks was set at 0.5 μm.

**FIGURE 2:**
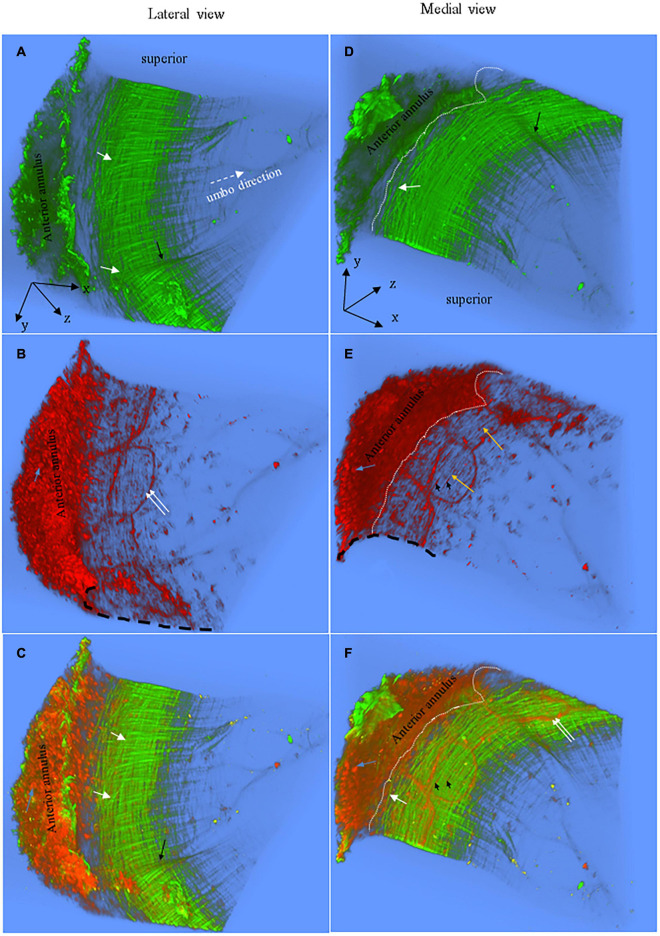
Representative 3D images of the anterior pars tensa-annulus unit reconstructed from SHG (green) and TPF (red) image stacks. **(A,D)** The lateral and medial view of the 3D collagen network. **(B,E)** The lateral and medial view of the 3D microstructure of the capillaries (double white arrows), elastic fibers (orange arrows), fibroblasts (black arrowheads) and annulus chondrocytes (blue arrows). **(C,F)** The merged images dedicate the 3D microstructure arrangement of the collagen network (green) with capillaries (double white arrows), fibroblasts (black arrowheads) and annulus chondrocytes (blue arrows). The 3D collagen network of the pars tensa notably exhibits a waveform contour [black arrows, panels **(A,C)**]. The white dash line draws approximately the boundary between the pars tensa and annulus. Field of the view: 509 × 509 μm^2^.

**FIGURE 3:**
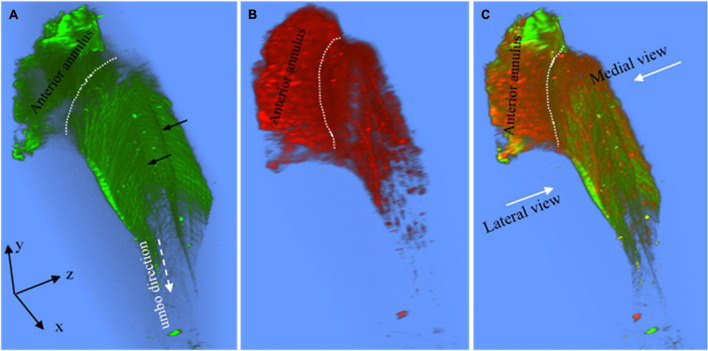
**(A)** The superior view of the 3D collagen scaffold (green) of the anterior pars tensa-annulus unit. The collagen scaffold in the pars tensa has a notable waveform contour (black arrows). **(B)** The corresponding TPF shows the capillary network and annulus chondrocytes of the parse tensa-annulus unit. **(C)** The merged image shows the capillary network (red) runs along the medial side and conforms to the collagen network (green). Field of the view: 509 × 509 μm^2^.

**FIGURE 4:**
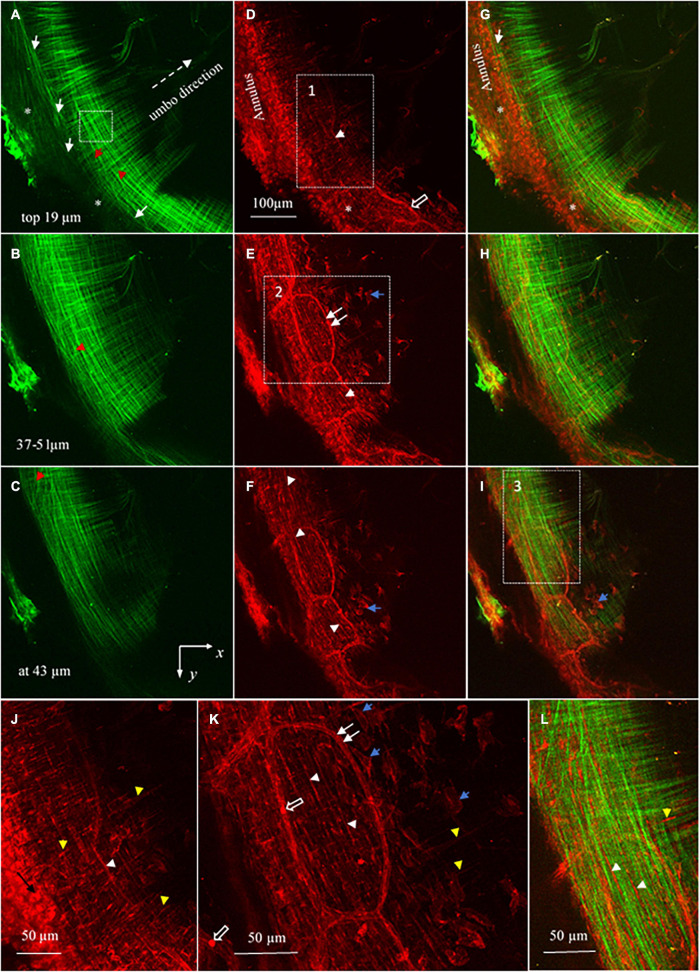
Maximum brightness images of the pars tensa annulus unit. **(A–C)** Maximum brightness images of SHG at different depths across the thickness of the pars tensa-annulus unit confirm that the collagen fibers constituting the parse tensa orient predominantly in the radial and circumferential direction, and the pars tensa and annulus are connected by collagen [* and white arrows, panels **(A,G)**]. Nevertheless, oblique and parabolic collagen fibers (red arrowheads) exist among the predominant radial and circumferential fibers. **(D–F)** The corresponding MBIs from TPF show the elastic fibers (white arrowheads), capillaries (double white arrows) with the hemoglobin (hollow arrow), vimentin positive cells (blue arrows) and annulus chondrocytes in the pars tensa-annulus unit. **(G–I)** The merged images highlight the integration of the collagen (green) with the chondrocytes, capillaries and vimentin cells (blue arrows) in the pars tensa-annulus unit. **(J)** An enlarged image extracted from region 1 in panel **(D)** showing in more detail the radial (yellow arrowheads) and circumferential (white arrowheads) elastic fibers and chondrocytes (black arrow) in the pars tensa–annulus unit. **(K)** An enlarged image extracted from region 2 in panel **(E)** showing in more detail the skeleton of vimentin positive cells (blue arrows), radial (yellow arrow heads) and circumferential (white arrow heads) elastic fibers and capillaries (double white arrows) with the hemoglobin (hollow arrows). **(L)** An enlarged image extracted from region 3 in panel **(I)** confirms that the pars tensa contains both the collagen fibers (green) and elastic fibers (white and yellow arrowheads) in the radial and circumferential direction.

**FIGURE 5:**
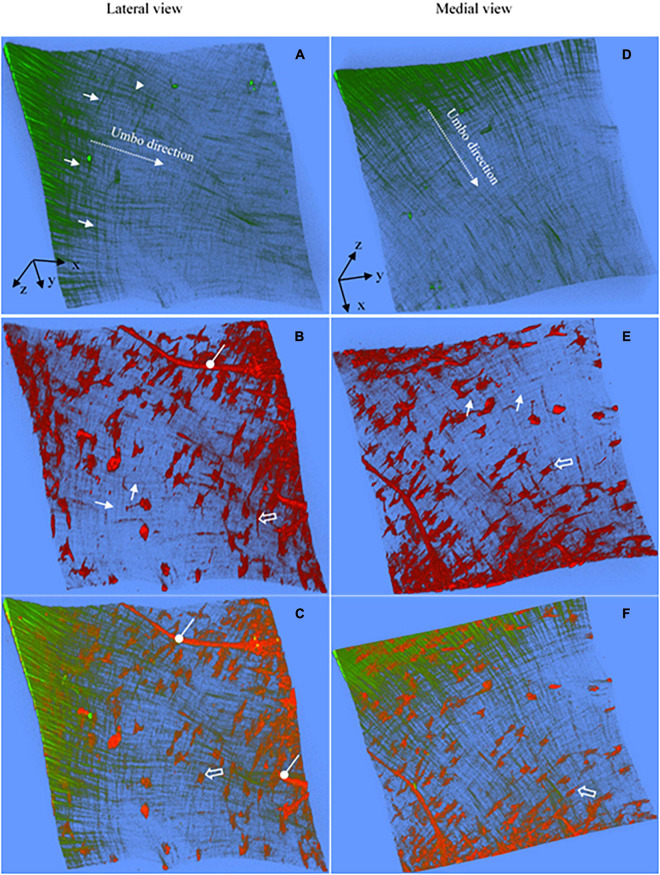
Representative 3D images reconstructed from the SHG and TPF imaging stacks of the pars tensa near the TM center. **(A,D)** The 3D collagen framework of the pars tensa near the TM center. **(B,E)** The corresponding TPF images show the capillaries (oval arrows), vimentin positive cells in various shapes (hollow arrow) and elastic fibers (white arrows) running in the radial and circumferential direction. **(C,F)** The merged images highlight the colocalization of the collagen (green) with the elastic fibers, vimentin positive cells and capillaries. The collagen fibers become progressively thinner approaching to the TM center so that the collagen network (green) appears as “a wave collagen flag” scattered with the vimentin positive cells and capillaries. Field of the view: 509 × 509 μm^2^.

**FIGURE 6:**
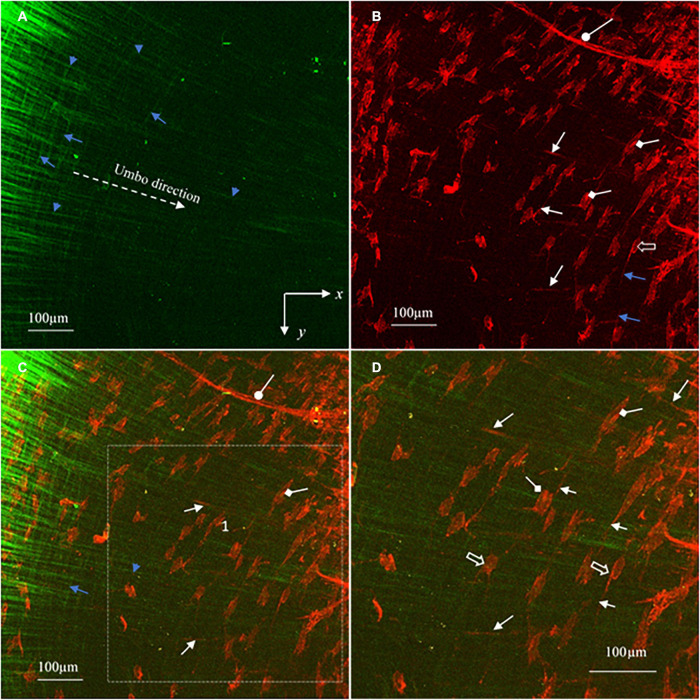
MBIs reconstructed from the image stacks in Figure 5 show in more detail the characteristics of the collagen (green), elastic fibers (white arrows), vimentin positive cells (hollow arrows), cell nuclei (diamond arrows) and capillaries (oval arrows) of the pars tensa near the TM center. **(A)** The collagen fibers gradually become finer approaching the TM center. **(B)** The corresponding TPF image shows the elastic fibers (white arrows), capillaries (oval arrow) and vimentin positive cells (hollow arrow) with the nuclei (diamond arrow). **(C)** The merged image dedicates the composition of the collagen fibers (green) with the cells, capillaries and elastic fibers. **(D)** An enlarged image extracted from region 1 of panel **(C)** highlight the characteristics of the fine collagen fibrils (green), elastic fibers (white arrows), vimentin positive cells (hollow arrow) and the cell nuclei (diamond arrow) of the pars tensa near the TM center. There is an apparent increase of the population of the vimentin positive cells near the TM center.

**FIGURE 7:**
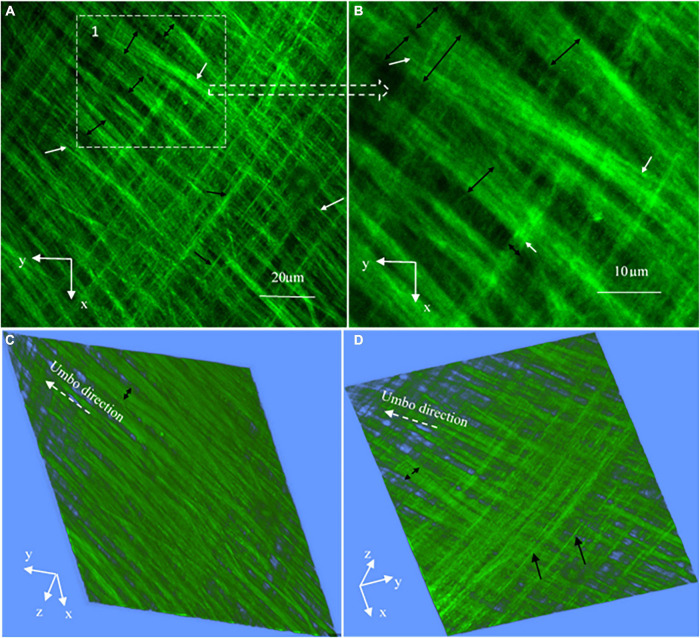
High magnification SHG images (at a 127 × 127 μm^2^ field of the view) at the periphery of the TM pars tensa (approximately at region 1 in Figure 4A). **(A)** The high magnification observations verify that the thick collagen fibers (double headed black arrows) of the periphery of the pars tensa are composed of the subclass collagen fibrils in the corresponding radial (white arrows) and circumferential (black arrows) direction. **(B)** An enlarged image extracted from region 1 of panel **(A)** shows in more detail that the collagen fibers (black double headed arrows) comprise the subclass collagen fibrils (white arrows). **(B,C)** The corresponding 3D images in the lateral **(C)** and medial **(D)** direction.

**FIGURE 8:**
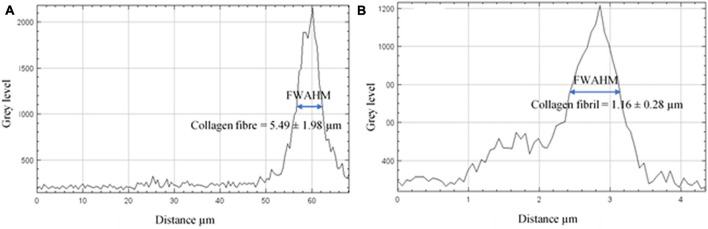
The gray level intensity distribution graphs across the collagen fibers and fibrils. **(A)** The FWAHM and gray level intensity distribution graph across the thickness of the collagen fibers were used to measure the thickness of the collagen fibers comprising the pars tensa. **(B)** The FWAHM and gray level intensity distribution graph across the thickness of the collagen fibrils were used to measure the thickness of the collagen fibrils.

The gray level intensity distribution graphs of the thickness of the collagen fibers and fibrils comprising the pars tensa were plotted, as shown in Figure 8. The full width at half maximum (FWAHM) intensity was used to determine the thickness of the collagen fibers and fibrils.

### Reconstructing 3D and Maximum Brightness Images

ImageJ (Rasband, 1997-2014) was used to reconstruct the SHG and TPF imaging stacks into 3D images to study the microstructure of the pars tensa-annulus unit (e.g., Figures 2, 3) and the tissue near the center of the TMs (Figure 5). For further studying the subtle internal features within the tissues, imaging stacks representing the microstructure of the full thickness tissues have also been optically subdivided into several smaller imaging stacks to reconstruct the maximum brightness images (MBI), as shown in Figures 4, 6. Here, an MBI is built by combining the pixels with the maximum intensity within a series of 2D images along the lateral-medial axis of the TM. Therefore, it represents a view of all the data in the 2D imaging series as if the imaging sequence were combined into a single image containing only the in-focus data.

### Quantify the Orientation of Collagen Constituting the Pars Tensa

Quantitative imaging analysis of the orientation of the collagen fibers comprising the pars tensa allows for greater consistency in describing the orientation characteristics over the subjective visual inspection. OrientationJ in ImageJ (Rezakhaniha et al., 2012) was used to quantify the orientation and coherency features of the collagen fibers constituting the pars tensa across the thickness from the lateral to medial direction. OreintationJ applies structural tensors to measure the gray level of pixels in the local neighborhood to determine the isotropic or anisotropic features in the image (Jähne, 1993; Jahne, 1997; Wu et al., 2017). Pixels with a high value indicate anisotropic features, whilst pixels with a low value represent isotropic features.

**FIGURE 9:**
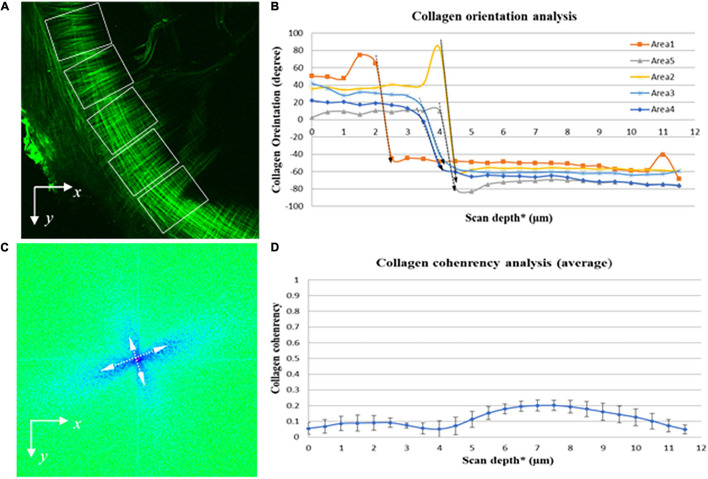
Quantitative imaging analysis of the orientation and coherency of the collagen fibers in the pars tensa from the lateral to medial side. **(A)** SHG images were divided into five regions of interest (dashed boxes) to quantify the orientation and coherency of the collagen fibers comprising the parse tensa from the lateral to medial side. **(B)** Collagen orientation analysis confirms across the TM thickness the collagen fibers comprising the pars tensa sharply change their orientation (dash arrows highlight). **(C)** FFT analysis confirms that the collagen fibers composing the pars tensa orient into two distinctive directions approximately perpendicular to each other. **(D)** The coherency analysis shows that the collagen fibers comprising the pars tensa have a very low coherency value just below 0.2. *The collagen scaffold in the pars tensa is in a shallow conical shape so that the scan depth has been used in the orientation and coherency analysis of the collagen fibers but the entire scan depth does not equal to the thickness of the pars tensa.

The SHG imaging stacks contain the spatial information of the collagen fibers comprising the pars tensa from the lateral to medial side. Therefore, OrientationJ has enabled quantifying (three-dimensionally) the orientation of the collagen fibers comprising the pars tensa across its thickness. Because the collagen scaffold in the pars tensa has a conical shape and the wall of the cone has a waveform contour (black arrows, Figures 2, 3), the collagen scaffold was subdivided into five regions of interest (ROI) (white boxes, Figure 9A) to conduct the fiber orientation and coherency analysis. The fiber orientation is shown in degrees from −90° to 90° in relation to the *x* axis (Figure 9A). The coherency has a value between zero and one. A value of one indicates the fibers are highly coherent whilst a value of zero indicates the fibers are extremely incoherent.

Fast Fourier transform (FFT) is a well-known mathematic procedure for efficiently identifying of the isotropic or anisotropic features in an image. FFT converts data in the spatial domain into the frequency domain, which makes it possible to efficiently identify the features in an image. FFT has also been used to objectively describe the orientation of the collagen fibers comprising the pars tensa (e.g., Figure 9C).

## Results

### 3D Microstructure of the Tympanic Membrane-Annulus Unit

Figures 2, 3 are representative 3D images of SHG (green) and TPF (red), showing the typical 3D collagen (green) and elastic (red) fibrillar network with the capillaries and cells (red) in the pars tensa-annulus (at region 1 of Figure 1B). The 3D TPF images (red) mainly illustrate the elastic fibrillar network (orange arrows in Figure 2E), the fibroblasts (black arrow heads in Figures 2E,F), capillaries (double white arrows in Figures 2B,F) and annulus chondrocytes in the pars tensa-annulus unit. Figures 2C,F dedicate the 3D composition of the collagen network (green) with the fibroblasts, capillaries and chondrocytes in the pars tensa-annulus unit. Unfortunately, the elastic fibers in the pars tensa are buried in the predominant collagen fibers in the merged images.

### Collagen Network

The typical 3D collagen network of the pars tensa-annulus unit is shown in Figures 2A,D. At the lateral side (Figure 2A), the collagen fibers comprising the pars tensa orient three-dimensionally in a primary direction radial toward the TM center. The radial fibers become thinner and thinner as they run toward the TM center. At the medial side (Figure 2B), the collagen fibers orient predominantly in a circumferential direction. These circumferential collagen fibers (green) originate from the annulus (Figure 2F). Nevertheless, the radial and circumferential fibers appear to braid with each other to form the 3D collagenous network of the pars tensa, whilst some oblique and parabolic collagen fibers (white arrows in Figures 2A,C,D,F, 3B, 4A,B) are observed among the predominated radial and circumferential fibers.

It is worth noting that the 3D collagen scaffold of the pars tensa demonstrates a waveform contour (long back arrows, Figures 2A,C,D, 3A). The capillary network (double white arrows in Figures 2B,F) is contained within the medial side of the pars tensa (Figures 2F, 3A) to conform to the contour of the collagen scaffold (green, Figures 3A,C).

### Elastic Fibers, Capillary and Cells

Without tissue stain, the TPF images (red) document the elastic fibers (orange arrows in Figure 2E, white arrow heads in Figures 4D–F,J–L), spindle fibroblasts (black arrow heads, Figures 2E,F), the capillary network (double white arrows, Figure 2B), vimentin positive cells (blue arrows, Figures 4D–F,J–L) and annulus chondrocytes (black arrow in Figure 4J) in the pars tensa-annulus unit. The spindle shape fibroblasts (black arrow heads, in Figure 2E) and circumferential elastic fibers (orange arrows) are within the pars tensa and connected into series compliant to the circumferential collagen fibers (Figure 4L). The capillaries (double white arrows, Figures 4E,K) were seen to contain hemoglobin (hollow arrows, Figures 4D–F,K). The TM annulus is a fibrocartilage tissue so that it contains massive chondrocytes.

The elastic fibers in the pars tensa can be seen in more detail in Figures 4D–F,J,K. As shown, the elastic fibers orient in both the radial (yellow arrowheads, Figures 4D,J) and circumferential (white arrowheads) direction. Figure 4L further confirms the co-existence of collagen (green) and elastic fibers in the pars tensa at the circumferential (white arrowheads) and radial direction (yellow arrowheads).

### The 3D Microstructure of the Pars Tensa Near the Tympanic Membrane Center

As shown in Figures 5A,B, as approaching the TM center (region 2, Figure 1B), the collagen network becomes much thinner, resembling “a waving collagenous flag” composed of fine collagen fibers running in the radial (white arrowheads, in Figure 5A) and circumferential (white arrows, Figure 5A) direction. The corresponding TPF images (Figures 5B,E) reveal the fine elastic fibers (white arrows), blood vessels (oval arrows) and vimentin positive cells in various shapes (hollow arrows). The integration of the 3D collagen network (green) with the vimentin positive cells, blood vessels and elastic fibers are shown in Figures 5C,F. Clearly, the tissues near the TM center contain more vimentin positive cells than those near the annulus.

The more detailed morphological characteristics of the micro components of the tissue near the TM center are shown in Figure 6. As confirmed in Figure 6A, the radial collagen fibers (blue arrowheads, Figures 6A,C) run continuously toward the TM center while they braid frequently with the circumferential fiber (blue arrows, Figures 6A,C). The corresponding TPF image (red, Figure 6B) shows in more detail the morphology of the blood vessels (oval arrow), fine radial (white arrows) and circumferential (blue arrows) elastic fibers, vimentin positive cells (hollow arrows) and the nucleus of the vimentin positive cells (diamond arrows). Figure 6D is an enlarged image from region 1 in Figure 6C that shows the detailed colocalization of the fine collagen fibers (green) with the elastic fibers (white arrows), vimentin positive cells (hollow arrows and the nucleus of vimentin positive cells (diamond arrows).

### Collagen Fibers and Fibrils

High magnification SHG images (Figure 7) acquired at the dashed square in Figure 4A have verified that the thick collagen fibers (double headed arrows) at the periphery of the pars tensa are made of the subclass collagen fibrils (white and black arrows). As shown in Figure 8, the collagen fibers are 5.49 ± 1.98 μm thick and the collagen fibrils are 1.16 ± 0.28 μm thick. The collagen fibrils orient in the radial and circumferential direction approximately compliant with the collagen fibers.

### Microstructural Integration of Pars Tensa and Annulus

The spatial microstructural integration between the pars tensa and annulus is shown in Figures 2C,F, 3C while the detailed connection mechanism between the two parts is shown in Figures 4A,D,G (indicated by ^∗^). The oblique and circumferential collagen fibers (white arrows, Figure 4A) are originated from the fibrocartilage annulus containing dense round chondrocytes (red, Figure 4G) and they are continued into the collagen network of the pars tensa. The radial collagen fibers of the pars tensa firmly connect to the oblique and parabolic/circumferential collagen fibers at the border with the annulus (^∗^ and white arrows, Figures 4A,G). The corresponding TPF images (Figures 4D,J) confirm the connection of the elastic fibers between the pars tensa and annulus.

### Quantitative Analysis of the Orientation of the Collagen Fibers

The quantitative analysis on the orientation of the collagen fibers comprising the pars tensa (Figure 9A) confirms that from the lateral to medial direction of the TMs the collagen fibers orient into two distinctive direction (long dashed arrows indicated in Figure 9B). This is consistent with the visual observations that across the thickness of the pars tensa the collagen fibers change from the radial orientation to the circumferential orientation. The FFT analysis also indicates that the collagen fibers comprising the pars tensa are largely oriented in two distinct directions approximately perpendicular to each other (double headed arrows in Figure 9C).

The coherency analysis (Figure 9D) shows that the collagen fibers comprising the pars tensa have a low coherency value below 0.2. This is consistent with the fact that the radial collagen fibers run approximately toward the TM center while the circumferential collagen fibers align approximately around the TM center. While the fibers in the two directions intensively braid with each other, neither the radial nor the circumferential fibers align parallel, resulting in the low coherency value. Also, the small portion of oblique and parabolic collagen fibers in the pars tensa has also contributed to the low coherency value.

## Discussion

Perforation or pathology of TMs is a serious medical condition leading to conductive hearing impairment and deafness if left untreated. Despite great efforts and advances in the research and development of collagen scaffold-based tissues engineering, 3D printing technologies and surgical interventions in the repairs of TMs, the outcomes are not yet satisfactory. This may be attributed to a lack of deep and accurate understanding about the 3D microstructure, composition and biology of the TMs at a close status to the native.

In this study, we demonstrated a 3D imaging technique for acquiring and examining the intrinsic 3D microstructure and composition of the lamina propria of hydrated mouse TMs in a full thickness. The study has brought new insights into the characteristics of the 3D microstructure and composition of the pars tensa-annulus unit. This information will be very useful for the development of cellular therapy and 3D printing technologies for regenerating a perforation or damaged TM.

Due to the close relationship between the microstructure and acoustic properties of TMs, the knowledge provided by this study will increase the understanding of the acoustic behavior and physiology of the TM. Also, the 3D imaging technique developed will offer a useful tool for studies of the disruption of the 3D microstructure and composition of TMs during otitis media and aging to the alteration of the acoustic function.

Collagen is the most abundant protein of the ECM of TMs determining the acoustic characteristics of the TMs. The microstructure and concentration of the collagen have a significant influence over the shape, mechanical properties and health status of the TMs (Fay et al., 2006). Using a 3D imaging technique that does not require tissue dehydration and sectioning, it has been confirmed in this study that the collagen fibers of the pars tensa form a 3D collagenous network constituted primarily of the radial fibers and circumferential fibers in the lateral and medial side respectively, with some oblique and parabolic collagen fibers among the predominant radial and circumferential fibers. This study has also confirmed that the thick collagen fibers at the periphery of TMs bordering the fibrocartilaginous annulus are made of the subclass collagen fibrils, and become finer toward the TM center. The findings are consistent with the previous studies (Secondi, 1951; Lim, 1968, 1970, 1995; Uno, 2000).

Although we have not examined the orientation and characteristics of the collagen fibers in respect to their influence on the acoustic properties of TMs, some studies have indicated that the radial and circumferential collagen fibers play individual roles in the sound transmission and acoustic behavior of the TMs (Fay et al., 2006). Therefore, the spatial orientation and characteristics of the collagen fibers in the pars tensa and the connection features of the collagen between the pars tensa and annulus revealed by this study will help studies and understanding of the sound propagation mechanism in TMs.

Relatively to the collagen, research on the elastin in the TMs is underrepresented in the literature (Lim, 1995). To our knowledge, there is not yet a study showing the form of the elastic fibers and the 3D characteristics of the elastic fibers with the collagen fibers, cells and capillaries in TMs. TPF imaging has been used by a number of studies for high resolution and label-free visualizing the 3D microstructure of the elastic fibers with cells in tissues (Zipfel et al., 2003; He et al., 2013b; Wu et al., 2020). The most striking discovery in this study is the 3D architecture of the elastic fibers with the collagen, and the close physical connection of the elastic fibers with the spindle fibroblasts in the pars tensa (Figures 2B,E). The close physical connection of elastic fibers with fibroblasts has also been found in studies of tendons (Pang et al., 2017), and has been suggested to be a possible way mediating the mechanotransduction between the fibroblasts and ECM of the tissue. Therefore, this study will open potential for studying the mechanism of hearing loss.

As a crucial building block of cellular membranes, blood vessel walls and ECM of many biological tissues, elastic fibers provide the tissues with the essential elasticity to recover quickly from dynamic strain while they also influence the physiology and disease states of many tissues (He et al., 2013b). Collagen is well known for its great tensile strength to keep and maintain the static shape of tissues while elastic fibers endow tissues with recoil properties to recover rapidly from dynamic deformation. Therefore, the coexistence and 3D morphological characteristics of the collagen and elastic fibers in the pars tensa may have important implications in studies of the acoustic behaviors and health status of TMs. The deterioration or absence of elastic fibers may lead to abnormal fatigue to accelerate the degradation of the acoustic properties of the TMs. Thus, the discovery of the elastic fibers as a coexisting fibrillar element to the predominant collagen fibers in the ECM of the pars tensa will increase the understanding of the biomechanics of TMs and degeneration mechanism of TMs with aging.

There are some factors that prevented observing sharply the elastic fibers and fibroblasts in the unstained status of the TMs using TPF, for accurately quantifying the orientation features of the elastic fibers. Firstly, the excitation efficiency for TPF decays dramatically with the increase of the excitation wavelength (Zheng et al., 2011; Wu et al., 2020). When the excitation wavelength increases from 600 to 700 nm, the excitation efficiency of the TPF drops by a factor of 10 (Zheng et al., 2011; Wu et al., 2020). In this study, the excitation laser available for the TPF imaging was at the near-infrared range and set at 890 nm. As a result, there was a significant decrease in the excitation efficiency of the TPF, and the intensity of the autofluorescence of the elastic fibers and fibroblasts in the images. Secondly, the MMP used in this study is only configured with a 25 X/NA 1.05 water immersion objective lens. The magnification power and numerical aperture of the objective lens is not high enough to reveal the skinning fibroblasts and elastic fibers in the TMs. Either the previous studies (Pang et al., 2017) or this present study (e.g., Figures 4J–L) have shown that the use of high magnification observations is crucial to discover the elastic fibers among massive volume of collagen fibers. Moreover, the fibroblasts, elastic and collagen fibrils are intensively tangled and integrated in the TMs, while the volume of the elastic fibers and fibroblasts is much less than that of the predominant collagen fibers. The elastic fibers and fibroblasts are buried in the larger volume of the collagen matrix, which makes them difficulty to be seen at low magnification observations (e.g., Figures 2C,F).

However, more recent studies have shown that the use of short wavelength excitation lasers at a visible range of 520 nm can improve greatly the excitation efficiency and signal to noise ratio in TPF imaging of unstained tissues (Wu et al., 2020). Therefore, future studies, using advanced multiphoton microscopy integrated short wavelength excitation laser and objective lenses with high magnification powers and numerical aperture, will lead to increase the visibility of the elastic fibers and fibroblasts among the abundant collagen fibers in TMs, allowing simultaneously quantifying the orientation characteristics of the collagen, elastic fibers and fibroblasts, and developing more sophisticated auto classification systems for scoring the physical conditions of TMs.

In addition, this study has found that the tissues near the TM center or umbo contain more vimentin positive cells than those near the annulus. This may explain why tissue cultures harvested from the umbo regions are more proliferative in the regrowth of vimentin positive cells than those from the annulus containing round chondrocytes (Liew et al., 2017). This is an important finding that could have implications for improving the efficacy of cellular therapeutic techniques for TM repairs.

Visualization of cells and tissue structure using high resolution imaging techniques is highly desirable in medical clinics and research because it provides a way to visually inspect the morphology of the cells for briefly determining the cell types and tissue health status. In this study, we reported the cells visualized by the 3D imaging technique using a visual inspection of the cell morphological characteristics. Therefore, it is crucial to carry out a study in future to accurately examine the phenotypes of the cells documented by the 3D imaging technique using advanced immunohistological and genetic tools.

## Data Availability Statement

The raw data supporting the conclusions of this article will be made available by the authors, without undue reservation.

## Ethics Statement

*Ex vivo* mouse TM samples used in this study were donated by other unrelated research in accordance with approvals from the Animal Ethics Committee at Southern University of Science and Technology, Shenzhen, China.

## Author Contributions

J-PW: conceptual design, supervision, data analysis and interpretation, and writing and revision of the manuscript. XY: sample preparation, imaging, experiment, and data analysis and writing the manuscript. YW and RZ: imaging. BS: quantitative imaging analysis, discussions, and critical revisions of the manuscript. RA, WZ, and YZ: discussion and revision of the manuscript. FC: conceptual design, supervision, and writing and revision of the manuscript. All authors contributed to the article and approved the submitted version.

## Conflict of Interest

The authors declare that the research was conducted in the absence of any commercial or financial relationships that could be construed as a potential conflict of interest.

## Publisher’s Note

All claims expressed in this article are solely those of the authors and do not necessarily represent those of their affiliated organizations, or those of the publisher, the editors and the reviewers. Any product that may be evaluated in this article, or claim that may be made by its manufacturer, is not guaranteed or endorsed by the publisher.
